# Dissociation of plasma oxyntomodulin levels from anthropometric measures and metabolic markers in women with polycystic ovary syndrome

**DOI:** 10.20945/2359-4292-2024-0451

**Published:** 2025-06-13

**Authors:** Rachel C. Damasceno, Flávia R. Oliveira, Ana Lúcia Cândido, Karina B. Gomes, Mariana F. Bizzi, Rosana C. Azevedo, Fábio V. Comim, Fernando M. Reis, Ana Luiza Lunardi Rocha

**Affiliations:** 1 Departamento de Obstetrícia e Ginecologia, Universidade Federal de Minas Gerais, Belo Horizonte, MG, Brasil; 2 Departamento de Clínica Médica, Universidade Federal de Minas Gerais, Belo Horizonte, MG, Brasil; 3 Departamento de Análises Clínicas e Toxicológicas, Universidade Federal de Minas Gerais, Belo Horizonte, MG, Brasil

**Keywords:** Polycystic ovary syndrome, oxyntomodulin, body mass index, visceral adiposity index, metformin

## Abstract

**Introduction:**

Oxyntomodulin (OXM) is a polypeptide hormone of the incretin family, which binds to the glucagon-like peptide 1 receptor, contributing to a reduction in caloric intake, an increase in energy expenditure, and weight loss in obese individuals. Polycystic ovary syndrome (PCOS) is a multifactorial condition characterized by reproductive and metabolic dysfunctions, with a high prevalence among overweight and obese women. This study aimed to investigate the correlation between clinical, anthropometric, endocrine, and metabolic variables and plasma OXM levels in women diagnosed with PCOS.

**Subjects and methods:**

This cross-sectional study included 20 women recently diagnosed with PCOS. Participants underwent screening that assessed body measurements, as well as serum/plasma lipids, fasting glucose, fasting insulin, irisin, and total testosterone levels. Plasma OXM concentrations were measured in duplicate using a commercial ELISA kit.

**Results:**

OXM levels were positively correlated with age (r = 0.493, p = 0.027); however, they showed no significant correlation with body mass index, waist circumference, lipid accumulation product index, visceral adiposity index, or hormones such as insulin, irisin, and testosterone. Furthermore, plasma OXM levels remained unchanged in a subgroup of patients treated with metformin for 60 days.

**Conclusion:**

These findings suggest that plasma OXM levels may not reflect body composition or insulin resistance in women with PCOS.

## INTRODUCTION

Oxyntomodulin (OXM) is a polypeptide hormone that belongs to the incretin family, secreted by intestinal enteroendocrine cells, specifically L cells, predominantly located in the terminal ileum and colon(^1^, ^2^). OXM is synthesized through the processing of pre-pro-glucagon to pro-glucagon, which is then processed by PCSK1 (Subtilisin/kexin type 1 proprotein convertase), culminating in the release of mature OXM(^3^). This hormone binds to the glucagon-like peptide 1 (GLP-1) receptor and the glucagon receptor(^4^), leading to the inhibition of glucagon secretion, a reduction in caloric intake, an increase in energy expenditure(^5^), and weight loss in obese patients (^6^). Abnormal secretion of incretins, including OXM, has been observed in individuals with type 2 diabetes mellitus (^7^).

Polycystic ovary syndrome (PCOS) is a multifactorial condition characterized by reproductive and metabolic traits and presents with various phenotypes. The classic form of PCOS includes hyperandrogenism (either clinical or biochemical), ovulation disorders with menstrual irregularity, and enlarged ovaries with numerous small antral follicles (^8^, ^9^). Obesity is an independent risk factor for PCOS; thus, lifestyle interventions that improve nutritional balance and reduce body adiposity are effective in reversing PCOS features and restoring spontaneous ovulation (^10^).

The regulation of incretins in women with PCOS remains poorly understood, despite the critical role of this peptide family in controlling food intake, energy metabolism, body composition, and insulin sensitivity (^11^). In a randomized controlled trial with 29 to 34 participants per group, the use of continuous oral contraceptive pills was associated with reduced plasma OXM levels in women with PCOS (^12^). The impact of other treatments on OXM levels in this population is still unknown.

The complex metabolic features of PCOS, changes in body composition, and androgen levels pose challenges in understanding how insulin resistance emerges and which adaptive mechanisms it evokes in the presence of the syndrome (^13^, ^14^). For instance, brown adipose tissue activity, which utilizes glucose and fatty acids to produce heat, is lower in individuals with PCOS; however, this reduction is related more to visceral adiposity than to hyperandrogenism (^15^).

Considering the lack of evidence concerning factors associated with OXM levels in women with PCOS, the aim of this study was to investigate which clinical, anthropometric, endocrine, and metabolic variables might correlate with OXM levels in this specific group of women. As a secondary objective, we evaluated OXM levels in a subgroup of patients before and after 60 days of treatment with metformin.

## METHODS

### Study design

We conducted a cross-sectional study with 20 women who had recently been diagnosed with PCOS at the outpatient facilities of two teaching hospitals in Belo Horizonte, Brazil. This study constitutes a secondary analysis of a subsample of participants from a larger study detailed elsewhere (^15^). The Ethics Committee of *Universidade Federal de Minas Gerais* approved the study (protocol no. 17127713.2.0000.5149), and all participating individuals provided written informed consent.

Participants underwent clinical consultations, physical examinations, and routine laboratory tests to exclude thyroid diseases, congenital adrenal hyperplasia, androgen-producing tumors, and hyperprolactinemia, following the Rotterdam diagnostic criteria for PCOS (^8^, ^9^). Additional exclusion criteria included pregnancy, the puerperium period, and oral glucose intolerance. None of the participants had used antiandrogenic drugs in the six months prior or other hormones in the two months preceding the study. However, a subgroup of patients (n = 5) underwent re-analysis 60 days after initiating treatment with 1,500 mg/day of metformin. The study population’s clinical characteristics are summarized in Table 1.

**Table 1:** Clinical characteristics of the study participants

Age (years)	32.6 ± 6.5
Body mass index (kg/m^2^)	27.9 ± 4.5
Waist circumference (cm)	90.3 ± 13.1
Lipid accumulation product index	36.9 ± 19.4
Visceral adiposity index	1.97 ± 1.11
Systolic blood pressure (mmHg)	114 ± 15
Diastolic blood pressure (mmHg)	73 ± 10
Triglycerides (mg/dL)	100 ± 34
HDL-cholesterol (mg/dL)	46 ± 12
Total cholesterol (mg/dL)	185 ± 27
Fasting glucose (mg/dL)	89 ± 7
Fasting insulin (µUI/mL)	13.8 ± 10.8
Irisin (ng/mL)	1116±899
Total testosterone (ng/dL)	36.9 ± 22.9

Data are expressed as means ± standard deviations.

### Anthropometrical assessment and laboratory tests

Participants, in light clothing and bare feet, had their body weight and waist circumference measured. The body mass index (BMI) was calculated using a standardized formula: weight in kilograms divided by height in meters squared. Combining waist circumference with serum triglycerides and/or HDL-cholesterol allowed for the calculation of the lipid accumulation product index and the visceral adiposity index, as detailed elsewhere (^14^). These indices serve as well-validated indicators of insulin resistance.

Blood samples were collected after a nine-hour overnight fast, between days 3 and 5 of a spontaneous bleeding episode, or on a random day in cases of amenorrhea. Laboratory assessments included serum lipids, fasting glucose and insulin levels, total testosterone, and plasma irisin levels (Table 1). Total testosterone was assessed using a competitive immunoassay (Vitros® system, Ortho Clinical Diagnostics, USA). Plasma irisin levels were measured in duplicate using a commercial ELISA kit (CUSABIO®, catalog no. CSB-EQ027943HU), adhering to the manufacturer’s instructions (^15^).

Plasma OXM levels were determined in duplicate with a commercial sandwich ELISA kit (Ansh Labs, catalog # AL-139), strictly in accordance with the manufacturer’s instructions. The assay’s detection limit is 0.25 pg/mL with 95% reliability, and it has a linear range of 3 to 300 pg/mL. The intra-assay and inter-assay coefficients of variation were <4% and <5%, respectively.

### Statistical analysis and power calculation

Variables were assessed using the D’Agostino and Pearson normality test and summarized as means ± standard deviations. Linear correlations were evaluated using Pearson’s coefficient, whereas repeated measures were analyzed through paired t-tests, always considering a p-value of <0.05 as statistically significant. The sample size was determined to allow for a significant estimation of correlation coefficients ≥0.6 with 80% statistical power and a 95% confidence level (^16^).

## RESULTS

**Figure 1:**
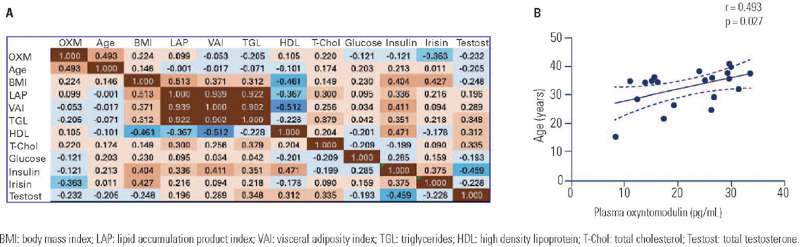
**(A)** Correlation matrix illustrating the relationship between clinical and biochemical variables that may be associated with plasma oxyntomodulin levels in women diagnosed with polycystic ovary syndrome. The data are presented as Pearson’s linear correlation coefficients, with the color intensity reflecting the degree of positive (red tones) or negative (blue tones) correlations. **(B)** A scatter plot that demonstrates the linear correlation between age and OXM levels.

BMI: body mass index; LAP: lipid accumulation product index; VAI: visceral adiposity index; TGL: triglycerides; HDL: high density lipoprotein; T-Chol: total cholesterol; Testost: total testosterone.

**Supplementary Figure 1:**
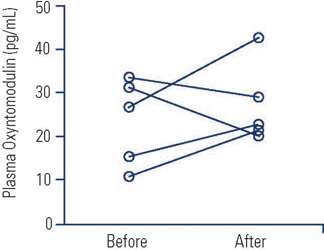
The individual changes in plasma oxyntomodulin levels in patients before and after 60 days of treatment with metformin, administered at a dose of 1,500 mg/day.

Plasma OXM levels ranged from 8.3 to 33.6 pg/mL, with a mean concentration of 22.1 pg/mL (95% confidence interval of 18.5-25.7 ng/mL). As illustrated in Figure 1, plasma OXM levels exhibited a positive correlation with age (r = 0.493, p = 0.027), but not with other biometric and biochemical variables. Notably, OXM levels displayed no correlation with BMI (r = 0.224, p = 0.342), lipid accumulation product index (r = 0.099, p = 0.748), visceral adiposity index (r = −0.053, p = 0.863), triglycerides (r = −0.205, p = 0.482), glucose (r = −0.121, p = 0.611), insulin (r = −0.121, p = 0.756), or testosterone (r = −0.232, p = 0.425). Among participants who received 1,500 mg/day of metformin, there was no significant change in plasma OXM levels after 60 days of treatment (p = 0.625; Supplementary Figure 1).

## DISCUSSION

This study investigated the clinical and metabolic characteristics of women with PCOS that might be closely associated with plasma levels of OXM. We found that OXM was positively correlated with age, but it was not associated with body measurements such as BMI or waist circumference, nor with any metabolic index or hormones like insulin or testosterone. Additionally, irisin, a myokine that stimulates insulin synthesis and secretion in response to glucose levels along with in-cretins (17), was not correlated with OXM. These findings suggest that OXM levels do not reflect body composition or insulin resistance in women with PCOS. The influence of age on OXM levels is a novel finding that warrants further investigation to confirm these results and to evaluate this phenomenon in different population groups before hypothesizing a physiological mechanism.

The study of incretin hormones such as GLP-1 and Glucose-Dependent Insulinotropic Polypeptide (GIP) in the context of PCOS has garnered significant interest (^11^, ^12^, ^18^). Conversely, OXM has primarily been investigated for its role in the treatment of obesity and diabetes (^19^, ^20^). The administration of subcutaneous OXM three times daily has led to significant weight loss in overweight and obese patients. This intervention also resulted in changes in adipokine levels consistent with weight loss and reduced caloric intake (^6^). However, given the short half-life of OXM and the challenges associated with multiple daily injections, analogues of OXM are now under development for clinical use in the treatment of diabetes (^21^).

The effects of lifestyle changes and hormonal contraceptives on circulating incretin levels have been explored in women with PCOS through secondary analysis of a randomized controlled trial (^12^). The group receiving oral hormonal contraception exhibited a significant suppression of plasma OXM levels, a response not induced by lifestyle interventions. Furthermore, the impact of metformin on the incretin axis was evaluated in PCOS patients over an eight-month period, showing an increase in GIP and GLP-1 levels post-intervention (^13^). Similarly, a study in non-diabetic men demonstrated an increase in GLP-1 levels after 14 days of metformin use compared to non-users (^22^). In mice, metformin administration increased GLP-1 levels, while GIP and peptide YY levels remained unchanged, suggesting that metformin does not directly stimulate the secretion of enteroendocrine L cells (^18^, ^23^). The absence of metformin’s effect on OXM observed in this study supports these hypotheses; nonetheless, our findings should be regarded as preliminary and interpreted with caution due to the small sample size evaluated after metformin use.

The present study has several limitations. The cross-sectional design prohibits causal or temporal inferences about the relationships between variables. Although the sample size was statistically adequate for the primary study objective, it was not large enough to allow for the subdivision into different PCOS phenotypes or BMI strata. Additionally, the participants were recruited from specialized clinics in teaching hospitals, which may not reflect the broader population seen in primary care settings or the community.

In conclusion, our findings suggest that, unlike other incretins (^11^), OXM is unrelated to body mass, adiposity, or insulin resistance in women with PCOS. Nonetheless, OXM or its pharmacological analogues may still play a role in treating obesity, insulin resistance, and glucose intolerance (^5^, ^6^), offering potential benefits for a significant subset of women with PCOS who also suffer from these comorbidities.

## Data Availability

the data that support the findings of this study are available from the corresponding author upon reasonable request.
